# Reconstructing a Network of Stress-Response Regulators via Dynamic System Modeling of Gene Regulation

**DOI:** 10.4137/grsb.s558

**Published:** 2008-02-10

**Authors:** Wei-Sheng Wu, Wen-Hsiung Li, Bor-Sen Chen

**Affiliations:** 1 Lab of Control and Systems Biology, Department of Electrical Engineering, National Tsing Hua University, Hsinchu, 300, Taiwan; 2 Department of Evolution and Ecology, University of Chicago, 1101 East 57th Street, Chicago, IL, 60637, U.S.A; 3 Genomics Research Center, Academia Sinica, Taipei, Taiwan

**Keywords:** dynamic system model, gene regulation, stress response, regulator

## Abstract

Unicellular organisms such as yeasts have evolved mechanisms to respond to environmental stresses by rapidly reorganizing the gene expression program. Although many stress-response genes in yeast have been discovered by DNA microarrays, the stress-response transcription factors (TFs) that regulate these stress-response genes remain to be investigated. In this study, we use a dynamic system model of gene regulation to describe the mechanism of how TFs may control a gene’s expression. Then, based on the dynamic system model, we develop the Stress Regulator Identification Algorithm (SRIA) to identify stress-response TFs for six kinds of stresses. We identified some general stress-response TFs that respond to various stresses and some specific stress-response TFs that respond to one specific stress. The biological significance of our findings is validated by the literature. We found that a small number of TFs is probably sufficient to control a wide variety of expression patterns in yeast under different stresses. Two implications can be inferred from this observation. First, the response mechanisms to different stresses may have a bow-tie structure. Second, there may be regulatory cross-talks among different stress responses. In conclusion, this study proposes a network of stress-response regulators and the details of their actions.

## Introduction

Single-celled organisms such as yeasts constantly face variable or even harsh external environments that threaten their survival or, at least, prevent them from performing optimally. Environmental changes can be of a physical or chemical nature such as temperature, oxidation, osmolarity, acidity and nutrient availability (Hohmann and Mager, 2003). The response mechanisms to stress are highly complex. One aspect of this cellular adaptation is the reorganization of gene expression. The gene expression program required for maintenance of the optimal cell physiology in one environment may be far from optimal in another environment. Thus, when the environmental condition changes abruptly, the cell must rapidly adjust its gene expression program to adapt to the new conditions (Gasch et al. 2000).

Large-scale reprogramming of gene expression can be revealed from genome-wide DNA microarrays, which measure the relative transcription levels of every gene in the yeast genome at any given moment, providing a snapshot of the genomic expression program (Gasch and Werner-Washburne, 2002). Exploring the dynamic nature of the yeast genome through time-course experiments can illuminate yeast stress responses. For example, Gasch et al. (2000) and Causton et al. (2001) used genome-wide expression analysis to explore how gene expression in yeast is remodeled over time as cells respond to heat shock, oxidative shock, osmotic shock, acidic stress, nitrogen depletion, amino acid starvation as well as other environmental stresses. They discovered that more than half of the genome is involved in responding to at least one of the investigated environmental changes. A set of genes (~10% of yeast genes), termed as the environmental stress response (ESR) genes or common environmental response (CER) genes, showed a similar drastic response to almost all of these environmental changes. Other gene expression responses appeared to be specific to particular environmental conditions. However, the regulators of these stress-response genes are not revealed from their studies. The complete network of stress-response regulators and the details of their actions remain to be investigated (Gasch et al. 2000).

Computational and statistical methods have been developed to identify plausible regulators of many cellular processes in yeast (Pilpel et al. 2001; Bar-Joseph et al. 2003; Segal et al. 2003; Middendorf et al. 2004; Yu and Li, 2005; Kim et al. 2006; Wu et al. 2006; Lin et al. 2007; Rokhlenko et al. 2007; Wu et al. 2007a; Wu et al. 2007b). When the time-course data of a system are available, using dynamic system modeling is more appropriate than using statistical approach because it can model the dynamic behavior of the system (Tegner et al. 2003; Chen et al. 2004; Chen et al. 2005; Chang et al. 2005; Chang et al. 2006). Therefore, in this paper we use a dynamic system model of gene regulation to describe the mechanism of how TFs may control a gene’s expression. Then, based on the dynamic system model, we develop the Stress Regulator Identification Algorithm (SRIA) to identify stress-response TFs for six different kinds of stresses. Our goal is to reconstruct a network of stress-response regulators and study the details of their actions.

## Methods

### Data sets and data preprocessing

Two kinds of data sets are used. First, for each gene in the yeast genome, the TFs that may regulate its expression are retrieved from the YEASTRACT database (Teixeira et al. 2006b). The regulatory associations between the target gene and the TFs are included if they are supported by published data showing at least one of the following experimental evidences: i) Change in the expression of the target gene due to a deletion (or mutation) in the gene encoding transcription factor; these evidences may come from detailed gene by gene analysis or genome-wide expression analysis; ii) Binding of the transcription factor to the promoter region of the target gene, as supported by band-shift, foot-printing or Chromatine ImmunoPrecipitation (ChIP) assays. However, the TF-gene regulatory association data are noisy. The genes whose expressions are affected by the mutation of a TF may not be the direct targets of that TF. Moreover, the genes that are bound by a TF identified by ChIP-chip experiments may not be regulated by that TF since TF binding does not necessarily mean regulation. Therefore, other independent data source such as gene expression or proteomic data should be used to filter out the noise inherent in the TF-gene regulatory association data.

In this study, we incorporate the gene expression data into our analysis. The genome-wide gene expression time profiles under various stress conditions such as heat shock, oxidative shock, osmotic shock, acidic stress, nitrogen depletion, and amino acid starvation are from Gasch et al. (2000) and Causton et al. (2001). Under each stress condition, samples for all genes in the yeast genome are collected at multiple time points. In order to reconstruct the missing values and avoid overfitting, the cubic spline method (Faires and Burden, 1998) is applied to interpolate some extra data points. (The missing values of the microarray data are those data points whose values are questionable due to the experimental defects. Therefore, these data points are not reported in the microarray data and regarded as the missing values.) In order to fit the dynamic system model in the linear scale, the microarray data are transformed from the log_2_ scale to the linear scale.

### Dynamic system model of gene regulation

We consider the transcriptional regulatory mechanism of a target gene as a system with the regulatory profiles of several TFs as the inputs and the gene expression profile of the target gene as the output. Owing to random noise and fluctuations at the molecular level, the transcriptional regulation of a target gene is described by the following stochastic dynamic equation

(1) $$y[t+1]=\left(\sum_{i=1}^{N}b_{i}\cdot x_{i}[t]+k\right)-\lambda \cdot y[t]+ɛ[t]$$

where *y*[*t*] represents the target gene’s expression profile at time point *t*, *N* denotes the number of TFs that may regulate the target gene’s expression, *b_i_* indicates the regulatory ability of TF*i*, *x_i_*[*t*] represents the regulatory profile of TF*i* at time point *t*, *k* represents the basal level induced by RNA polymerase II, *λ* indicates the degrading effect of the present state value *y*[*t*] on the next state value *y*[*t*+1] and *ɛ*[*t*] denotes a stochastic noise due to the modeling error and measuring error of the target gene’s expression profile. *ɛ*[*t*] is assumed to be a Gaussian noise with mean zero and unknown standard deviation *σ*. The biological meaning of Equation (1) is that *y*[*t*+1] (the target gene’s expression value at the next state) is determined by ∑*_i_*_=1_^N^*b_i_*·*x_i_*[*t*] *k* (the production effect of the *N* TFs at the present state and RNA polymerase II) and −*λ*·*y*[*t*] (the degradation effect of the target gene at the present state).

It has been shown that TF binding usually affects gene expression in a nonlinear fashion: below some level it has no effect, while above a certain level the effect may become saturated. This type of binding behavior can be modeled using a sigmoid function (Chen et al. 2004; Chen et al. 2005; Chang et al. 2005; Chang et al. 2006; Wu et al. 2006; Wu et al. 2007a). Therefore, we define *x_i_*[*t*] (the regulatory profile of TF*i* at time point *t*) as a sigmoid function of *z_i_*[*t*] (the gene expression profile of TF*i* at time point *t*):

(2) $$x_{i}[t]=f(z_{i}[t])=\frac{1}{1+\text{exp}\left[-r(z_{i}[t]-A_{i})\right]}$$

where *r* denotes the transition rate of the sigmoid function and *A_i_* denotes the mean of the gene expression profile of TF*i*.

### Estimating the parameters of the dynamic system model

Using the TF-gene regulatory association data from the YEASTRACT database and gene expression data from Gasch et al. (2000) and Caustion et al. (2001), we can estimate the parameters of the dynamic system model in Equation (1). We rewrite Equation (1) to the following regression form:

(3) $$\begin{array}{l} y[t+1]=\left[x_{1}(t)\ldots x_{N}(t)1-y[t]\right]\cdot \left[\begin{array}{c} b_{1}\\ \vdots \\ b_{N}\\ k\\ \lambda \end{array}\right]+ɛ[t]\\ \equiv \varphi [t]\cdot \theta +ɛ[t] \end{array}$$

where *φ*[*t*] = [*x*_1_[*t*] … *x_N_*[*t*] 1 − *y*[*t*]] denotes the regression vector and *θ* = [*b*_1_ … *b_N_* *k λ*]*^T^* is the parameter vector. *N* is the number of the TFs that may regulate the target gene’s expression and can be inferred from the TF-gene regulatory association data.

From the gene expression data, it is easy to get the values of {*x_i_*[*t_v_*], *y*[*t_v_*]} for *i* ∈{1, 2, …, *N*}, *v* ∈ {1, 2, …, *M*}, where *M* is the number of the time points of a target gene’s expression profile. Equation (3) at different time points can be put together as follows

(4) $$\left[\begin{array}{c} y[t_{2}]\\ y[t_{3}]\\ \vdots \\ y[t_{M}] \end{array}\right]=\left[\begin{array}{c} \varphi [t_{1}]\\ \varphi [t_{2}]\\ \vdots \\ \varphi [t_{M-1}] \end{array}\right]\cdot \theta +\left[\begin{array}{c} ɛ[t_{1}]\\ ɛ[t_{2}]\\ \vdots \\ ɛ[t_{M-1}] \end{array}\right]$$

For simplicity, we can further define the notations *Y*, Φ and *e* to represent Equation (4) as follows

(5) Y=Φ·θ+e

The parameter vector *θ* can be estimated by the maximum likelihood (ML) method as follows (Johansson, 1993)

(6) $$\hat{\theta }={\left(\Phi^{T}\Phi \right)}^{-1}\Phi^{T}Y$$

### Stress Regulator Identification Algorithm (SRIA)

After constructing the dynamic system model of gene regulation as in Equation (1) and estimate the parameter vector as in Equation (6), we are now ready to identify the stress-response TFs for six kinds of stresses. We develop Stress Regulator Identification Algorithm (SRIA) to do this task. SRIA can be divided into the following three steps:

**Step 1:** Identification of stress-response genes

The first step is to find out all the genes in the yeast genome that respond to a specific stress (e.g. heat shock, oxidative shock, osmotic shock, acidic stress, nitrogen depletion and amino acid starvation). A gene is said to respond to a specific stress if more than two time points of its gene expression profile measured under that specific stress are induced or repressed by more than three folds compared to that of the unstressed condition (see Supplementary Table 1 for details).

**Step 2:** Identification of stress-response TFs

For each stress-response gene identified in Step 1, we retrieve all TFs that may regulate its expression from the TF-gene regulatory association data. Knowing this information enables us to construct the dynamic system model of the transcriptional regulation of the stress-response gene as in Equation (1). Then we apply the ML method to estimate the parameter vector *θ* =[*b*_1_ … *b_N_* *kλ*]*^T^* of the model and get the ML estimate *θ̂* = [*b̂* … *b̂_N_* *k̂ λ̂*]*^T^* as in Equation (6). Since *b_i_* stands for the regulatory ability of TF*i*, a small absolute value of *b_i_* means that TF*i* only has a negligible effect on the stress-response gene’s expression. Therefore, this TF-gene regulatory association may be a false positive and should be eliminated from our analysis. On the other hand, a large absolute value of *b_i_* means that TF*i* has a large regulatory effect on the stress-response gene’s expression. We regard TF*i* to be a stress-response TF if its regulatory ability *b_i_* is statistically significantly different from zero (i.e. |*b_i_*| ≫ 0). The test statistic $t={\hat{b}}_{i}/(s\sqrt{u_{ii}})$, a *t*-distribution with degree of freedom (*M* − 1) − (*N* + 2) is used to assign a *p*-value for rejecting the null hypothesis *H*_0_: *b_i_* *=* 0, where *u_ii_* is the *i* th diagonal element of the matrix (Φ*^T^*Φ)^−1^ and $s=\sqrt{{\left(Y-\Phi \cdot \hat{\theta }\right)}^{T}(Y-\Phi \cdot \hat{\theta })/((M-1)-(N+2))}$ is an unbiased estimator of *σ* (the standard deviation of the stochastic noise *ɛ*[*t*]) (Mendenhall and Sincich, 1995). The *p*-value computed by the *t*-distribution is then adjusted by Bonferroni correction to represent the true α level in the multiple hypothesis testing (Mendenhall and Sincich, 1995). Then, TF*i* is said to be involved in response to a specific stress if the adjusted *p*-value *p_adjusted_* ≤ 0.01 (see Supplementary Table 1 for details).

**Figure 1:**
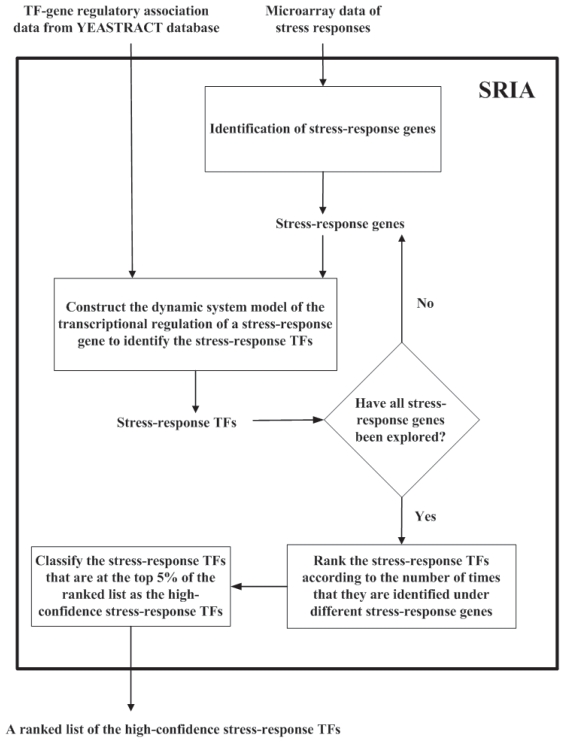
The flowchart of SRIA.

**Step 3:** The stress-response TFs identified in Step 2 are ranked according to the number of times that they are identified under different stress-response genes. The first TF in the list is the one that is identified with the largest number of times. The TFs that are at the top 5% of the ranked list are classified as the high-confidence stress-response TFs. Finally, we output a ranked list of the high-confidence stress-response TFs for each of the six different kinds of stresses. The flowchart of SRIA could be seen in Figure 1.

## Results

### Stress-response TFs

We identified the TFs that respond to heat shock, oxidative shock, osmotic shock, acidic stress, nitrogen depletion, and amino acid starvation, respectively. Table 1 shows the high-confidence stress-response TFs for each of the above six stresses. The identified stress-response TFs can be divided into two categories. The first category is the well-known stress-response TFs with solid literature evidence that directly indicates involvement of these TFs in response to that specific stress. The second category is the novel stress-response TFs that have only partial or no literature support. (The partial literature support means that these TFs are predicted by pervious studies as plausible stress-response TFs but still need further verification.) We found that 38% (13/34 counting multiplicity) of the identified stress-response TFs belongs to the first category, indicating the effectiveness of SRIA. In addition, 52% (11/21 counting multiplicity) of the second category has partial literature support, revealing the predictive power of SRIA. Therefore, the novel stress-response TFs (Arr1, Ifh1, Rpn4 and Sok2) that have no literature evidence yet are worthy of experimental verification.

## Biological validation of predictions

Now we discuss in detail our predictions that are supported by experimental evidence in the literature.

### Heat shock

The predicted heat shock TFs Msn2, Msn4, Rpn4, Yap1 and Cad1 have solid or partial literature evidence. First, Msn2 and Msn4 bind DNA at stress response element (STRE) and activate many STRE-regulated genes in response to many stresses such as heat shock, oxidative shock and osmotic shock (Cherry et al. 1998). Second, it has been demonstrated that the heat shock TF Hsf1 co-ordinates a feed-forward gene regulatory circuit for *RPN4* heat induction (Hahn et al. 2006). Third, Yap1 is known to induce the expression of *GSH1* and *GSH2* to synthesize glutathione in heat shock response (Suqiyama et al. 2000). Fourth, overexpression of Cad1 is known to increase thermo-tolerance of a cell under starvation conditions (Cherry et al. 1998).

### Oxidative shock

The predicted oxidative shock TFs Sfp1, Yap1, Rpn4 and Rap1 have solid or partial literature evidence. First, Sfp1 is known to regulate ribosomal protein (RP) gene expression in response to various stresses such as oxidative shock and osmotic shock (Marion et al. 2004). Second, Yap1 is known to regulate genes that respond to oxidative shock. For example, Yap1 regulates *TRX2*, a cytoplasmic thioredoxin isoenzyme of the thioredoxin system which protects cells against oxidative stress (Güldener et al. 2005). Third, *RPN4* promoter contains a Yap1 binding site (YRE) which is responsible for *RPN4* induction in response to oxidative stress. (Hahn et al. 2006). Fourth, it is known that Rap1 signaling is required for suppression of Ras-generated reactive oxygen species and protection against oxidative stress (Remans et al. 2004).

### Osmotic shock

The predicted osmotic shock TFs Msn2, Msn4, Sfp1 and Yap1 have solid or partial literature evidence. First, the msn2-msn4 double deletion mutants exhibit higher sensitivity to severe osmotic stress, indicating Msn2 and Msn4 are involved in response to osmotic stress (Cherry et al. 1998). Second, Sfp1 regulates RP gene expression in response to various stresses such as oxidative shock and osmotic shock (Marion et al. 2004). Third, the *YAP4* gene, previously shown to play a role in response to hyperosmotic stress, is regulated by the transactivators Yap1 and Msn2 (Nevitt et al. 2004).

### Acidic stress

The predicted acidic stress TFs Msn2, Msn4 and Rpn4 have solid or partial literature evidence. First, it is known that *RGD1* is activated at low pH. The transcription level at low pH was demonstrated to depend on the STRE box located in the *RGD1* promoter. The general stress-activated TFs Msn2 and Msn4 were shown to act mainly on the basal *RGD1* transcriptional level in normal and stress conditions (Gatti et al. 2005). Second, under acute herbicide 2,4-dichlorophenoxyacetic acid (2,4-D) stress, 14% of the yeast transcripts suffered a greater than twofold change. Most of the up-regulated genes in response to 2,4-D are known targets of Msn2, Msn4 and Rpn4 (Teixeira et al. 2006a).

### Nitrogen depletion

The predicted nitrogen depletion TFs Sfp1, Ifh1, Fhl1, Rpn4 and Rap1 have partial literature evidence. First, ribosomal protein (RP) genes in eukaryotes are coordinately regulated in response to growth stimuli and environmental stress, thereby permitting cells to adjust ribosome number and overall protein synthetic capacity to physiological conditions. Sfp1, Fhl1 and Ifh1 are known to regulate RP gene expression in response to nutrient depletion (Cherry et al. 1998; Marion et al. 2004; Güldener et al. 2005). Second, on solid growth media with limiting nitrogen source, diploid budding-yeast cells differentiate from the yeast form to a filamentous, adhesive and invasive form. Both low availability of nitrogen and a solid growth substrate are required to induce diploid filamentous-form growth. It is known that Rpn4 regulates filamentous growth, indicating that Rpn4 is involved in response to nitrogen depletion (Prinz et al. 2004). Third, Rap1 is known to be involved in the regulation of nitrogen, sulfur and selenium metabolism (Güldener et al. 2005).

### Amino acid starvation

The predicted amino acid starvation TFs Gcn4, Rap1 and Sfp1 have solid or partial literature evidence. First, Gcn4 is a well-known transcriptional activator of amino acid biosynthetic genes in response to amino acid starvation (Cherry et al. 1998). Second, *HIS4* encodes an enzyme that catalyzes the histidine biosynthesis and Rap1 is required for the rapid increase in the *HIS4* mRNA level following amino acid starvation (Devlin et al. 1991). Third, ribosomal protein (RP) genes in eukaryotes are coordinately regulated in response to growth stimuli and environmental stress, thereby permitting cells to adjust ribosome number and overall protein synthetic capacity to physiological conditions. Sfp1 is known to regulate RP gene expression in response to nutrient depletion (Cherry et al. 1998; Marion et al. 2004).

## Discussions and Conclusions

In this study, we use a dynamic system model of gene regulation to describe the mechanism of how the stress-response TFs may control a stress-response gene’s expression. Based on the dynamic system model, we develop the Stress Regulator Identification Algorithm (SRIA) to identify the stress-response TFs for each of six kinds of stresses. Some general stress-response TFs that respond to various stresses and some specific stress-response TFs that respond to a specific stress are identified. For example, we found the well-known general stress-response TFs Msn2, Msn4, Yap1, Rpn4 and Sfp1, consistent with the results of *Saccharomyces* Genome Database (SGD) (Cherry et al. 1998). Besides, the well-known heat shock TF Cad1, nitrogen depletion TF Fhl1 and amino acid starvation TF Gcn4 are identified. The ability to identify these well-known stress-response TFs validates the effectiveness of SRIA.

**Figure 2:**
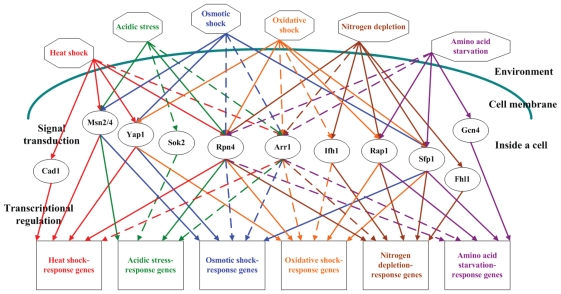
The network of stress-response regulators in yeast Environmental stresses are represented by octagons, stress-response TFs are represented by ellipses and stress-response genes are represented by rectangles. Solid (or dashed) lines indicate the known (or predicted) regulatory relationships among environmental stresses, stress-response TFs and stress-response genes. Heat shock responses are in red, acidic stress responses are in green, osmotic shock responses are in blue, oxidative shock responses are in orange, nitrogen depletion responses are in brown and amino acid starvation responses are in purple.

**Figure 3:**
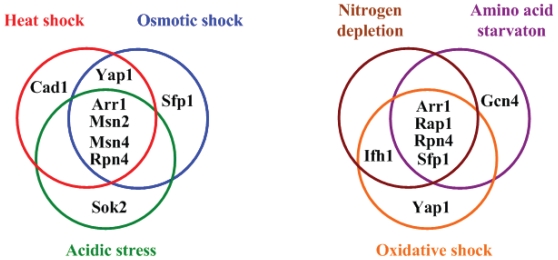
Regulatory cross-talks among different stress responses The cellular responses to heat shock, osmotic shock and acidic stress may have regulatory cross-talks. They all trigger TFs Arr1, Msn2, Msn4 and Rpn4. Moreover, the cellular responses to oxidative shock, nitrogen depletion and amino acid starvation may have regulatory cross-talks. They all trigger TFs Arr1, Rap1, Rpn4 and Sfp1.

SRIA identified 12 distinct TFs (Arr1, Cad1, Fhl1, Gcn4, Ifh1, Msn2, Msn4, Rap1, Rpn4, Sfp1, Sok2 and Yap1) to be in response to at least one of the six stresses under study (see Figure 2). This indicates that a small number of TFs may be sufficient to control a wide variety of expression patterns in yeast under different stresses. Two implications can be inferred from this observation. First, the response mechanisms to different stresses may have a bow-tie structure (Csete and Doyle, 2004). As shown in Figure 2, the core stress-response TFs make up the ‘knots’ of a bow tie, facilitating the fan in of a large variety of environmental stresses through signal transduction pathways and fan out of an even larger variety of stress-response proteins through activating stress-response target genes. Actually, approximately two-thirds of the yeast genome (about 3600 genes) is involved in responding to the changes in environment (Causton et al. 2001). Second, there may exist regulatory cross-talks among different stress responses (see Figure 3). We found that heat shock, osmotic shock and acidic stress all can trigger TFs Arr1, Msn2, Msn4 and Rpn4, indicating that these three stresses may share a similar stress response mechanism. In addition, we found that oxidative shock, nitrogen depletion and amino acid starvation all can trigger TFs Arr1, Rap1, Rpn4 and Sfp1, indicating crosstalks among the cellular responses to these three stresses. The fact that different stress response mechanisms share some, but not all, of their regulators suggests a higher level of modularity of the yeast stress response network (Segal et al. 2003).

**Figure 4:**
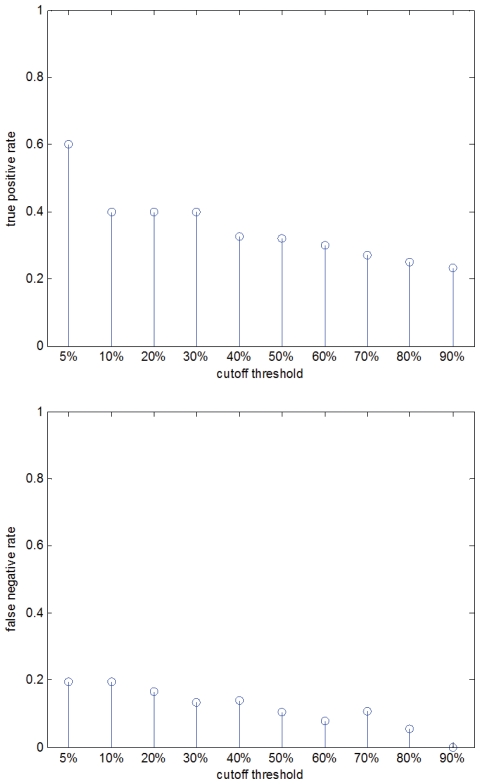
Statistics of the performance of SRIA using different cutoff thresholds The true positive and false negative rates of SRIA using different cutoff thresholds are shown. When the cutoff threshold equals 5%, SRIA has the best performance in terms of the tradeoff between maximizing the true positive rate and minimizing the false negative rate to find out the known amino acid starvation TFs. (The true positives are those known amino acid starvation TFs that are found by SRIA and the false negatives are those known amino acid starvation TFs that are not found by SRIA).

In Step 3 of SRIA, only those stress-response TFs that are at the top 5% of the ranked list are classified as the high-confidence stress-response TFs and reported as the final result. The reason for choosing only the top 5% of the ranked list is that when the cutoff threshold equals 5%, SRIA has the best performance in terms of the tradeoff between maximizing the true positive rate and minimizing the false negative rate to find out the known amino acid starvation TFs (see Figure 4 for details).

The response mechanisms to stress are highly complex. They require a complex network of sensing and signal transduction leading to the adaptation of cell growth and proliferation along with the adjustments of the gene expression program, metabolic activities and other features of the cell (Hohmann and Mager, 2003). This study focused on the regulation of gene expression and proposed a network of stress-response regulators and their details of actions. Thus, it provides a starting point for understanding the adaptation mechanisms that yeast uses to survive some of the environmental stress conditions, experienced in the wild. We believe that as more gene expression data are accumulated, in combination with data from other whole-organism approaches, novel computational algorithms such as SRIA have the potential to construct a dynamic picture of the integrated cellular response of yeast cells to environmental changes.

## Figures and Tables

**Table 1:** The high-confidence TFs in response to each of the six different stresses The high-confidence TFs in response to heat shock, oxidative shock, osmotic shock, acidic stress, nitrogen depletion and amino acid starvation are shown. The TFs are in red (blue) if there exist solid (partial) experimental evidence showing that they are involved in the same stress as we predicted.

Heat shock TFs	Oxidative shock TFs	Osmotic shock TFs	Acidic stress TFs	Nitrogen depletion TFs	Amino acid starvation TFs
**Arr1**	**Sfp1**	**Arr1**	**Msn2**	**Sfp1**	**Arr1**
**Rpn4**	**Rpn4**	**Sfp1**	**Msn4**	**Rpn4**	**Gcn4**
**Msn2**	**Arr1**	**Rpn4**	**Arr1**	**Rap1**	**Rpn4**
**Msn4**	**Yap1**	**Yap1**	**Rpn4**	**Arr1**	**Sfp1**
**Yap1**	**Rap1**	**Msn2**	**Sok2**	**Fhl1**	**Rap1**
**Cad1**	**Ifh1**	**Msn4**		**Ifh1**	
